# Human Factors and Technological Characteristics Influencing the Interaction of Medical Professionals With Artificial Intelligence–Enabled Clinical Decision Support Systems: Literature Review

**DOI:** 10.2196/28639

**Published:** 2022-03-24

**Authors:** Michael Knop, Sebastian Weber, Marius Mueller, Bjoern Niehaves

**Affiliations:** 1 Department of Information Systems University of Siegen Siegen Germany

**Keywords:** artificial intelligence, clinical decision support systems, CDSS, decision-making, diagnostic decision support, human–computer interaction, human–AI collaboration, machine learning, patient outcomes, deep learning, trust, literature review

## Abstract

**Background:**

The digitization and automation of diagnostics and treatments promise to alter the quality of health care and improve patient outcomes, whereas the undersupply of medical personnel, high workload on medical professionals, and medical case complexity increase. Clinical decision support systems (CDSSs) have been proven to help medical professionals in their everyday work through their ability to process vast amounts of patient information. However, comprehensive adoption is partially disrupted by specific technological and personal characteristics. With the rise of artificial intelligence (AI), CDSSs have become an adaptive technology with human-like capabilities and are able to learn and change their characteristics over time. However, research has not reflected on the characteristics and factors essential for effective collaboration between human actors and AI-enabled CDSSs.

**Objective:**

Our study aims to summarize the factors influencing effective collaboration between medical professionals and AI-enabled CDSSs. These factors are essential for medical professionals, management, and technology designers to reflect on the adoption, implementation, and development of an AI-enabled CDSS.

**Methods:**

We conducted a literature review including 3 different meta-databases, screening over 1000 articles and including 101 articles for full-text assessment. Of the 101 articles, 7 (6.9%) met our inclusion criteria and were analyzed for our synthesis.

**Results:**

We identified the technological characteristics and human factors that appear to have an essential effect on the collaboration of medical professionals and AI-enabled CDSSs in accordance with our research objective, namely, training data quality, performance, explainability, adaptability, medical expertise, technological expertise, personality, cognitive biases, and trust. Comparing our results with those from research on non-AI CDSSs, some characteristics and factors retain their importance, whereas others gain or lose relevance owing to the uniqueness of human-AI interactions. However, only a few (1/7, 14%) studies have mentioned the theoretical foundations and patient outcomes related to AI-enabled CDSSs.

**Conclusions:**

Our study provides a comprehensive overview of the relevant characteristics and factors that influence the interaction and collaboration between medical professionals and AI-enabled CDSSs. Rather limited theoretical foundations currently hinder the possibility of creating adequate concepts and models to explain and predict the interrelations between these characteristics and factors. For an appropriate evaluation of the human-AI collaboration, patient outcomes and the role of patients in the decision-making process should be considered.

## Introduction

### Background

From a global perspective, many health care systems face comprehensive challenges that affect how care is delivered to society. In this regard, several factors increasingly strain care structures, processes, and the actors involved. For instance, demographic changes and the overall aging of society raise age-related health issues and demands [1,2] and introduce further case complexity; for example, in the form of comorbidity [3]. Simultaneously, a shortage of personnel and medical expertise can be discerned in many—often remote and rural—regions, caused by the low attractiveness of jobs in care due to inappropriate compensation and high workload [4], the attractiveness of urban areas and structures [5], the absence of young graduates willing to establish new or continue existing practices [6], or the trend toward centralized care facilities, inter alia [7]. As a result, larger catchment areas develop for providers who have to cope with deficient and inequitably distributed first-hand access to care [8]. Further, on a societal level, detrimental access to care can marginalize lower socioeconomic groups, as a study from the United States suggests [9], impeding the maintenance of comprehensive and inclusive care. Considering the increasing complexity of medical care on the one hand and the decreasing time and personnel resources on the other hand, the need to actively support clinicians at the point of care is growing.

### Clinical Decision Support Systems

Representing a promising and widely adopted technology to render processes and decisions more efficient, so-called clinical decision support systems (CDSSs) are software applications capable of catalyzing and informing the process of decision-making of medical professionals [10]. Although applications exist that target the decisional processes of patients, often called decision aids [11] or patient decision support interventions [12], the clinical use of CDSSs remains the primary domain for decision support. Here, the evaluation of performance, adoption, effectiveness, and impact on patient outcomes advances, but still lacks comprehensive approaches [10], including an analysis of relationships among technological characteristics, continual use, and effects on diagnosis and treatment. Nevertheless, the potential of CDSSs to support diagnostic processes leads to their use in other contexts of medicine; for example, primary care [13], and in several different disciplines, from emergency medicine [14] and dermatology [15] to radiology [16]. Aside from diagnostic purposes, CDSSs are used to detect possible inadequate prescriptions of medication [17] or to simulate different treatment strategies and their impact on patient outcomes [18]. Until today, CDSSs have had partial nonadoption for numerous reasons; for example, workflow disturbances or trust deficits, and their adoption is linked to many different factors concerning technology and human-technology interaction [19,20]. In particular, the subjective perception of and attitude toward the CDSS remains a crucial predictor of adoption [21]. This is because the CDSS surpasses the preferably objective description of medical information (eg, in electronic health records) and interprets this information to support clinical interventions [19]. Meanwhile, the comparability of CDSS among different contexts is difficult because of the already-mentioned variation in user groups (patients, physicians, nurses, etc), medical domains (clinical care, primary care, etc), medical disciplines (dermatology, radiology, etc), and purposes (diagnosis, prescription, treatment, etc).

Owing to technological innovations, health care technologies, including CDSSs, are increasingly enabled by artificial intelligence (AI) [22]. The first evaluation of an AI-enabled CDSS promises increased performance and accuracy compared with a conventional CDSS [23]. In addition, clinicians and experts in the field generally expect simplification of organizational processes, such as patient flows, with the advent of AI [24]. Defined as a technology’s capability to work in a way that a human perceives as intelligent [25], AI is used on various occasions with regard to CDSS, such as risk prediction for medical complications [26] and adverse drug effects [27]. However, a rigorous and consistent definition of AI is challenging. Therefore, we followed Helm et al [28] and Schuetz and Venkatesh [29] on their emphasis on the adaptive characteristics of AI, meaning that AI-enabled CDSSs are learning entities that change over time while considering their environmental conditions. Consequently, these systems are not deterministic and may provide different outputs from the same input at different times [30]. Compared with medical professionals, AI-enabled systems can outperform human ratings or predictions; for example, concerning the classification of dermal lesions and proliferation [31]. Regarding the adoption of AI-enabled systems in general, ongoing research reports several concerns indicated by clinicians. Although the fear of being replaced appears to depend on the level of knowledge about the concept of AI that the clinicians possess [32], studies report that clinicians fear being biased by the recommendations of AI, resulting in overconfidence and harmful consequences for patients [33]. In addition, clinicians are concerned that AI might increase the threat of data breaches and the associated risks for patients’ privacy, as well as legal consequences resulting from treatment errors [34]. Nevertheless, current research suggests an ambivalent perception of AI. Considering the aforementioned concerns and potential hindrances for adoption, clinicians assume that AI-enabled systems might save time and improve the continuous monitoring of patients [35]. Furthermore, research has highlighted that only a few clinicians comprehend the variety of applications of AI and its conceptual nature [34,35]. Differences in the perception of AI; for example, regarding the fear of being replaced [36], emphasize the subjectivity of clinicians’ attitude toward AI.

Considering the ambiguity of concerns regarding clinicians’ attitudes toward AI, the mentioned hindrances of CDSS adoption and the similarity between concerns associated with AI and CDSS (eg, biased decision-making, legal consequences, or fear of being replaced), an AI-enabled CDSS might actually increase the relevance of perceptive and subjective factors for adoption and their interplay with technological characteristics. During the process of development and evaluation of AI-enabled CDSS, it became apparent that the potential benefits for clinical performance and treatment quality are maximized by human–AI collaboration, rather than by human-AI competition [31]. However, owing to the interactive and adaptive nature of AI-enabled CDSS, traditional theories and models to explain the use and adoption of these systems forfeit their power to explain and predict a successful collaboration between AI and human beings [29,37]. Specific factors regarding AI-enabled technology and human actors such as dermatologists, radiologists, and other medical professionals are emphasized to influence the relationship among them, including the explainability or understandability of the system [38], its purpose [39], and the resulting trust a human actor perceives in the system [40]. Considering that factors related to the subjective attitude and perception of clinicians, such as trust, already impact the adoption of non-AI CDSS [21,41,42], we argue that the advent of AI-enabled systems increases the importance of specific factors that are not exclusively bound to technological characteristics. Considering the already investigated hindrances impeding the adoption of CDSS by clinicians [43,44], the lack of a sound theoretical basis, or the reliance on traditional theoretical approaches within ongoing research [45], the need for a review of AI-specific factors influencing the collaboration between AI and human actors has increased.

### Human-AI Interaction and Collaboration

To understand the dyadic relationship between humans and AI, it is necessary to understand key concepts and their interrelations. Although many researchers use the term interaction [46], literature defining what interaction means is seldom. Hornbæk et al [46] showed that there is no common definition and identified 7 concepts of interaction that highlight different perspectives. However, the human–computer interaction framework of Li and Zhang [47] shows that interaction can be generally understood as a process of using a technology for a task in a specific context. In turn, collaboration etymologically stems from the term *collaborare* which means *work with*. As the origin reveals, collaboration can be understood as a joint effort in which a common goal is pursued. From our perspective, collaboration is thus a successful interaction with an adaptive AI-enabled system. Under the assumption that both human and AI-enabled systems are not error-free, a human-AI collaboration is effective when errors are prevented. In this context, a key driver of such effective collaboration is that medical professionals perceive the system as trustworthy (ie, a certain level of trust) for the tasks to be done and accept it. Trust is a complex psychological construct that is described as the will to make oneself vulnerable [48]. If a party considers another party to be trustworthy, the relationship is in turn determined by the perception of the other parties’ attributes of ability (the legitimacy of a system’s recommendation for a specific decision), benevolence (the accordance of a human actor’s and the system’s intention and motivation to do good), and integrity (the accordance of a human actor’s and the system’s superordinate values) [40]. Nevertheless, it remains unclear how system design can influence the perception of trustworthiness and what human traits foster the propensity to trust.

### Objectives

The objective of this study is to summarize the factors influencing effective collaboration between medical professionals and AI-enabled CDSSs. Capturing these factors is essential for medical professionals, management, and technology designers to reflect the adoption, implementation, and development of AI-enabled CDSSs [48,49]. Further, we seek to explore what specific outcomes are used to evaluate successful collaboration between humans and AI-enabled CDSSs (performance, effectiveness, impact on patient outcomes, etc) and the theoretical foundations on which they are based. Finally, the comparison between factors that are associated with AI-enabled CDSSs and those associated with CDSSs not enabled by AI appears to be important in evaluating the extent to which the current literature has already reflected the uniqueness of human-AI collaboration.

## Methods

### Overview

We conducted a narrative review to summarize the current literature regarding our specific objectives [50]. In the following, we report the search for relevant literature to meet our objective, its selection, and its synthesis to counteract the subjectivity of our results [50]. We selected 3 different meta-databases to search for studies that met our research objective. We defined our search strategy in accordance with the relatively broad scope of our study [51]. To report our results, we followed the PRISMA (Preferred Reporting Items for Systematic Reviews and Meta-Analyses) guidelines for reviews [52]. Through our initial search, we identified 1161 studies by screening titles and abstracts, of which 100 (8.61%) satisfied our inclusion criteria. Through a backward search, we identified another study that was included in our full-text assessment, resulting in 101 articles assessed for eligibility. Finally, 6.9% (7/101) of studies were included in our synthesis of results.

### Databases

We included the databases PubMed, PsycInfo, and Business Source Complete for our literature review. PubMed indexes >5000 journals in the fields of medicine, health care, and related disciplines. We used PubMed, in particular, to gather information about the clinical effectiveness and implementation of AI-enabled CDSSs. PsycInfo contains >2000 journals from behavioral and social science research. We searched PsycInfo to examine the psychological dimensions of AI-enabled CDSSs and decisional processes. Finally, we scanned results from Business Source Complete, containing >1000 journals in the field of business sciences, to obtain insights regarding our objective from an economic and procedural perspective.

### Study Selection

**Figure 1:**
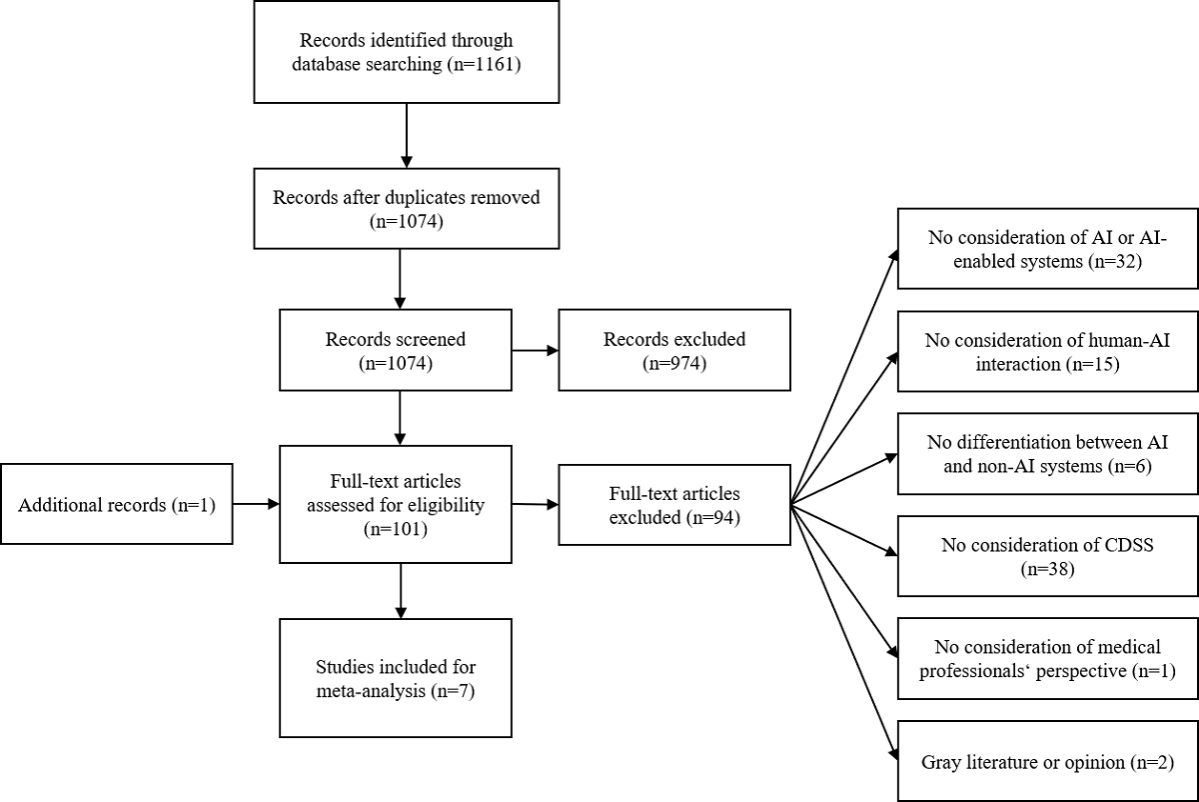
PRISMA (Preferred Reporting Items for Systematic Reviews and Meta-Analyses) flowchart. AI: artificial intelligence; CDSS: clinical decision support system.

We combined 2 different sections of search terms (AND conditions). The first section represented the technologies associated with the objective of our research *(AI OR artificial intelligence OR machine learning OR cognitive computing OR intelligent agent OR decision support OR recommendation agent)*. The second section reflected the interactional dimension of human-AI collaboration (trust* OR acceptance OR *agreement OR consent OR compliance OR congruency OR collaboration OR resistance). We included articles published in English over the last 10 years. To select relevant literature, 2 authors (MK and SW) independently screened titles and abstracts to exclude articles that did not involve AI-enabled technology (see the definition in the *Clinical Decision Support Systems* section) and those that were not related to health care or medicine. The inclusion and exclusion criteria were discussed in detail before the screening. In addition, to familiarize themselves with the procedure, an initial sample of 100 entries was screened. A high level of agreement was achieved, and disagreements were resolved through discussion between the 2 authors (MK and SW). In the remaining papers, only a few borderline cases were discussed until consensus was reached, and both the authors (MK and SW) finally came to the same result. In the full-text screening, articles that did not involve AI or AI-enabled systems (n=32), did not consider the interaction between the human actors and AI-enabled systems (n=15), did not distinguish between AI-enabled and non–AI-enabled CDSSs (n=6), did not involve CDSS (n=38) or the perspective of medical professionals (n=1), or appeared to be gray literature or opinion (n=2) were excluded. Detailed documentation of the exclusion process for full-text screening is provided in Multimedia Appendix 1, where all excluded studies and the reasons for exclusion are presented. The selection of relevant literature is represented through a PRISMA flowchart (Figure 1). If articles were eligible, we summarized and reported the specific factors influencing effective collaboration.

## Results

### Overview

On the basis of our study selection, 7 studies were included in our final synthesis. From our perspective, this result stems from the fact that many studies of AI-enabled CDSSs (1) compare solely the diagnostic accuracies of human raters and those of AI-enabled systems and (2) focus on technological characteristics and the development of these systems, but do not discuss their effects on the interaction or collaboration between technology and human actors. Therefore, most (5/7, 71%) of the included studies reflected on the relevance of specific characteristics or factors by contemplating the objective from a meta-perspective (Table 1).

**Table 1:** Summary of study characteristics included in our review.

Study	Type of study	Context	Focal point of interest
Cabitza et al [53]	Narrative review	Clinical care; health care (general); clinicians; no specific purpose	Trust
Felmingham et al [54]	Narrative review	Clinical care; dermatology; physicians; diagnostics	Mortality or morbidity
Gomolin at al [55]	Narrative review	Clinical care; dermatology; physicians; diagnostics	Explainability
Reyes et al [56]	Narrative review	Clinical care; radiology; physicians; diagnostics	Trust; explainability
Jeng and Tzeng [57]	Quantitative study	Clinical care; health care (general); physicians; diagnostics	Intention
Tschandl et al [31]	Quantitative study	Clinical care; dermatology; physicians; diagnostics	Performance
Asan et al [30]	Narrative review	Clinical care; health care (general); clinicians; no specific purpose	Trust

### Factors Influencing Collaboration

#### Technological Characteristics

This study addresses different dimensions or steps in the development, implementation, and adoption of an AI-enabled CDSS. The technological characteristics of these systems, that is, the abilities and attributes of technology that are defined by their design [58], are described as meaningful determinants for the way the interaction between the system and the human actor is shaped. For instance, Cabitza et al [53] concluded that a “truthful, reliable, and representative” system needs high-quality data based on which it is trained. Similarly, Asan et al [30] argued that the development of a “healthy trust relationship” with algorithmic decision-making relies on the thoughtful design of system characteristics. In general, the resulting performance of the system and its ability to explain or justify its conclusions appear to be strong predictors of a positive relationship [30,31,54-56]. Reyes et al [56] defined the explainability of an AI-enabled system as the ability to ensure that a human actor understands “the link between the features used by the machine learning system and the prediction.” In current literature, explainability and transparency of a system are often used interchangeably [54] or in the sense that transparency appears to be a superordinate category of explainability [30]. Closely linked to a system’s ability to explain its internal processes is the resulting effect on human actors with respect to the subjective interpretability of the given information [55].

Furthermore, Tschandl et al [31] argue that the output of an AI-enabled CDSS in its dimensions of simplicity, granularity, and concreteness might affect the final decision of clinicians; the better an AI-enabled system’s output is adapted to the situational context of its use, the more precise the overall diagnostic performance of the AI and humans (eg, clinicians facing multiclass diagnostic problems are supported by AI-based multiclass probabilities). In addition, a study mentioned the importance of usability and user satisfaction for effective human-AI collaboration [53] but does not provide a definition in the context of AI-enabled CDSSs.

#### Human Factors

In addition, social (eg, trust), psychological (eg, personality traits), and cognitive characteristics (eg, cognitive biases) of a human actor affecting their interaction with technology, that is, human factors [59], appear to be meaningful prerequisites for the relationship between systems and actors as well. Asan et al [30], Tschandl et al [31], Felmingham et al [54], and Jeng and Tzeng [57] argued that the clinical experience of medical professionals is a highly important factor in determining the interaction and performance of human-AI collaboration. In general, these studies show that less experienced physicians benefit the most from AI-enabled CDSSs and attain a higher overall diagnostic accuracy, whereas an experienced physician’s diagnostic accuracy differs little or not at all. In addition, Asan et al [30] and Felmingham et al [54] argue that technological experience and even the personality of medical professionals are important factors for medical professionals’ decision-making processes, although no study has yet investigated their effect on the collaboration between AI-enabled CDSSs and human actors. Furthermore, Asan et al [30] and Felmingham et al [54] mentioned that the relationship between the system and human actor can be disrupted by several cognitive biases affecting collaboration at different times, that is, confirmation bias, anchoring effect, overconfidence, availability bias, framing effect, premature closure, and automation bias. Already known from medical decision-making in general, cognitive biases alter rational processes of medical professionals, resulting in erroneous diagnostics and treatments [60]. Because of biased thinking in decisional processes and the variety of biases occurring at different times in these processes, AI-enabled CDSSs are prone to be affected by these biases [54].

Among the included studies, a human actor’s trust in an AI-enabled CDSS appeared to be another important factor that directly influenced the quality of collaboration and adoption of technology. For instance, Cabitza et al [53] argued that a lack of trust might result from different technological characteristics and their situational fit but always negatively impacts the overall performance of the human-AI team. Reyes et al [56] hypothesized that the comprehensible explainability of a system ensures a high level of trust, including a system’s ability to explicate its learning process and essential or most effective determinants for its prediction, as well as adequate and situational visualization of its internal processes. Felmingham et al [54] argued that trust is created through an interactional process between AI and humans. Accordingly, Asan et al [30] also highlighted the interdependency of human factors and system features as constituting factors of trust. However, Asan et al [30] argued that maximizing trust should not be the ultimate goal, as AI also has its limitations in that blind trust could lead to undesirable consequences. Instead, system designers should establish mechanisms that encourage reciprocal skepticism, create healthy trust relationships, and maximize the accuracy of clinical decisions. From this perspective, trust is highly dependent on the personality of the human actor, system design, and cognitive biases that might emerge in the collaboration. The reported technological characteristics and human factors influencing effective AI-human collaboration are summarized in Table 2.

**Table 2:** Technological characteristics and human factors influencing and shaping the relationship and collaboration between AI-enabled clinical decision support systems (CDSSs) and human actors.

Parameters		Definition	Study
**Technological characteristics**			
	Training data quality	Information used for training of AI-enabled CDSSs to create a truthful, reliable, and representative system	[53]
	Performance	The accuracy and reliability of an AI-enabled CDSS	[30,55]
	Explainability or transparency	An AI-enabled CDSS' ability to ensure that a human actor understands the processes that lead to the prediction and the prediction itself	[30,31,54-56]
	Adapted output or adaptability	The degree to which an AI-enabled CDSS fits into a specific context or environment according to the subdimensions simplicity, granularity, and concreteness	[31]
**Human factors**			
	Medical expertise	The degree of medical experience of a human actor within the context of collaboration with an AI-enabled CDSS	[30,31,54,57]
	Technological expertise	The degree of technological experience of a human actor with regard to an AI-enabled CDSS	[30,54]
	Personality	A medical professional’s attributes and characteristics that influence the interaction with AI-enabled a CDSS	[54]
	Cognitive biases	The cognitive processes that alter rational decision-making and perceptions of an AI-enabled CDSS	[30,54]
	Trust	The subjective impression of a medical professional that an AI-enabled CDSS is truthful and reliable	[30,53,54]

### Evaluation of Medical Outcomes

Of the 7 included studies, only 1 (14%) study mentioned the interrelation between an effective human-AI collaboration and primary clinical outcomes. Reviewing an AI-enabled CDSS for skin cancer diagnostics, Felmingham et al [54] mentioned the possible impacts of these systems on a patient’s morbidity and mortality associated with skin cancer in general. Other studies described secondary outcomes, such as a system’s mathematical accuracy [55] or behavioral intentions to use a CDSS [57]. No study investigated the impact of technological characteristics or human factors on primary clinical outcomes.

### Theoretical Foundation of Research

Of the 7 included studies, only 1 (14%) study mentioned the theoretical foundations on which implications for practice are based explicitly. Jeng and Tzeng [57] derived hypotheses for their empirical investigation from the unified theory of acceptance and use of technology, which is a technology acceptance theory widely adopted to explain the intention to use technology and the subsequent use behavior [61]. An important predecessor in this theoretical model is social influence (ie, “...the degree to which an individual perceives that important others believe he or she should use the new system” [61]). However, based on their results, Jeng and Tzeng [57] discarded their theoretical assumption about social influence affecting clinicians’ intentions to use a CDSS. Felmingham et al [54] discussed the role of cognitive biases in decisional processes involving AI-enabled CDSSs. Nevertheless, Felmingham et al [54] did not explicitly mention the origin of cognitive biases in the prospect theory by Kahneman and Tversky [62].

## Discussion

### Principal Findings

**Figure 2:**
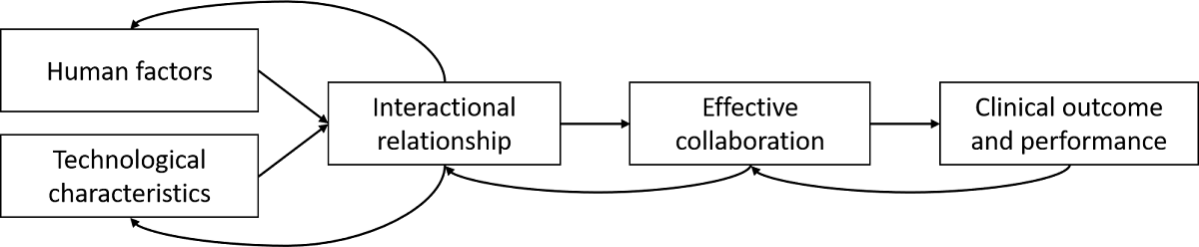
Steps and elements of reciprocal processes of human–artificial intelligence collaboration.

Our results show that only a few (7/101, 6.9%) studies have already broached the issue of individual factors influencing effective collaboration between a human actor and an AI-enabled CDSS. Although unique considerations with regard to these systems appear; for example, the important role of trust [30,53], scarce empirical evidence exists for the relational structure of essential factors or characteristics. In addition, many studies did not describe the involved system and its characteristics extensively, enabling differentiation between AI and non–AI-enabled systems accurately [42]. Therefore, we argue that a more thorough description of the involved system and its characteristics is highly relevant for future research as it lays the foundation for comparing different systems and their effectiveness. Nevertheless, in the process of reviewing the literature, we were able to differentiate between factors primarily associated with technological structures and functions (technological characteristics), and those primarily associated with human actors’ psychological or perceptual attributes (human factors). Both technological characteristics and human factors influence the nature of the interaction between human actors and AI-enabled CDSSs. Interestingly, some technological characteristics and human factors appear to be antecedents of interaction; for example, the personality of medical professionals [54], whereas others appear to be effects of an interaction [53]. Therefore, as suggested by Felmingham et al [54], it can be assumed that human factors and technological characteristics are mutually dependent and together shape the interaction between human actors and AI-enabled CDSSs. As described in the *Background* section, the shape of an interaction between human actors and AI and their resulting interactional relationship can be considered a condition for successful collaboration. However, the foundation for evaluating an AI-enabled CDSS differs, in accordance with current research addressing non-AI CDSS [20]. Studies from our results discussed the accuracy or mathematical performance of systems, adoption by medical professionals, sustainability and congruency of interaction, and the effects on patient outcomes to be relevant for evaluation. Although the effectiveness of a collaboration between human actors and AI currently depends on the context and objective of a system [53], the paradigm of medicine clearly dictates the final evaluation of a CDSS by its ability to improve primary and secondary outcomes of patients [22]. As AI-enabled systems are characterized by their adaptive nature [29], processes of individual interaction and collaboration are likely to be iterative and reciprocal and will change and be refined over time. Figure 2 summarizes this process based on our results and can be considered a proposed descriptive framework for human-AI collaboration.

When comparing our results to research concerning medical professionals’ interactions with non-AI CDSS, high correspondence can be noted. Khairat et al [20] mentioned workflow fit (adaptability), computer literacy (technological expertise), trust, a general optimistic attitude of clinicians (personality), and clinical expertise (medical expertise) as important factors for effective adoption. In addition, Khairat et al [20] reported usability and perceived usefulness as determinants. As perceived usefulness needs further concretization within the context of AI-enabled systems [53], usability might generate only minor relevance for AI-enabled CDSSs, as these systems are based on automated processes of use and integrate human-like ways to communicate (eg, natural language processing for voice control) [29]. In contrast, the explainability of a CDSS appears to be a technological characteristic that strongly influences the collaboration between humans and the system, whether it is enabled by AI or not [63]. However, differentiation of explainability and related terms such as understandability, interpretability, and transparency has not yet been completed, and the impact of explainability on other relevant factors, including trust, has not yet been empirically verified [63]. In general, it is not clear how and if technological characteristics and human factors influence other specific aspects of collaboration between human actors and AI-enabled CDSSs. For instance, studies suggest that high clinical expertise influences overall collaborative performance [54] but does not hypothesize possible explanations. Clinical expertise might be associated with a lack of trust in these systems, overconfidence biases, or the fact that these systems are sometimes less accurate than experienced physicians.

Furthermore, other studies involving non-AI CDSSs have emphasized the essential role of trust in the effective interaction between humans and the system. Trust is a multidimensional construct. A lack of trust might result from reservations regarding the mathematical accuracy or appropriateness of a system or the purpose of a system in improving patient outcomes [64]. As our literature review reveals the importance of the technological accuracy of AI-enabled CDSSs, research focusing on trust in human-like technology has shown that ability, benevolence, and integrity are essential prerequisites for sustainable adoption [37,40]. However, only 14% (1/7) of the included studies highlighting trust in its role in successful collaboration defined the actual meaning of trust [30], and none of the included studies paid attention to the prerequisites. Considering the inconsistent definition of trust in technology [48,65], future research might reveal important prerequisites for trust within the interaction between human actors and AI-enabled technologies. In addition, the relationship between trust in AI-enabled CDSSs and improvement in clinical outcomes requires further investigation.

Findings from our literature as well as ongoing research concerning non-AI patient decisional aid suggest that a stronger theoretical foundation for the interaction between human actors and CDSSs is important [66]. Felmingham et al [54] already demonstrated that cognitive biases, originating from the prospect theory, might decisively impact effective collaboration, that is, the tendency to confirm assumptions already made rather than falsify them, known as confirmation bias [67], might distort the relationship between medical professionals and AI-enabled CDSS in the sense that they might not accept a different opinion except their own. In contrast, relying on automated information instead of vigilantly seeking and interpreting information, known as automation bias [68], might actually cause the unreflected acceptance of suggestions made by a CDSS. Therefore, to discuss suitable theoretical foundations, it might be helpful to further explicate and structure the aforementioned nontransparent relations of different constructs, factors, and characteristics influencing decision-making and collaboration. In addition, problems originating from the application of traditional technology-centered theories (such as the technology acceptance model or unified theory of acceptance and use of technology) on AI-enabled decision-making might lead to inappropriate results [29,69]. Theories concerning the trust-based adoption of human-like technology [40] promise to encounter these deficits by emphasizing the interactional components of technology adoption and use.

### Limitations

Our study had some limitations. As some studies derived their conclusions about collaboration between AI-enabled CDSSs and human actors from studies of CDSSs not enabled by AI or assigned results from non-CDSSs to CDSSs, reasoning about interrelations between different technological characteristics and human factors is preliminary and requires further investigation. Although our results fit well with the current findings about the uniqueness and specific nature of human-AI interaction, very few (7/101, 6.9%) studies, of which most were narrative reviews, were included because of our innovative and novel objective as well as the specific context. This may be a result of our relatively narrow search, which could be extended by explicating the related constructs and prerequisites of trust. Explorative empirical studies based on suitable theoretical foundations might yield frameworks and models to structure future research on AI-enabled CDSSs, as our study primarily provides an orientation about relevant individual characteristics and factors. The consideration of environmental influences (eg, organizational policies or culture [30] and patients’ views [70]) on AI-supported decisional processes for medical care is vital for a comprehensible understanding but cannot be provided within the scope of our review.

### Conclusions

We extracted the technological characteristics and human factors relevant for effective collaboration between medical professionals and AI-enabled CDSSs. Although most of the findings from previous research on non–AI-enabled CDSSs are in accordance with our results, the weighting of specific factors might change with AI-enabled systems. The adaptive and increasing human-like nature of AI-enabled CDSSs emphasizes the time sensitivity and reciprocity of decisional processes that should ultimately lead to an improvement in care. Cognitive biases may occur at any time during these processes, varying the effectiveness of collaboration. Explainability remains an essential prerequisite for interaction, and the expertise and personalities of medical professionals have come into focus. In addition, trust between humans and the system emerges as a central aspect of decisional support, whereas the interrelations among these facets still need to be investigated. Concepts such as shared decision-making justify the integration of patients’ demands and wishes, an important factor for medical care, and its role in human-AI collaboration is yet underrepresented. Currently, it is unclear how these concepts can be integrated into AI-enhanced decisional processes and to what extent medical decisions with the help of the CDSS are influenced by the subjective meaning and understanding of diagnoses or treatments by patients. In addition, as several studies have measured the effectiveness of collaboration by means of other parameters, primary and secondary patient outcomes should be considered in future research.

As described earlier, modern health care structures are under increasing pressure. Involved medical professionals face immense workloads per capita, and the supply of personnel declines. Because these structures form the initial access points for most citizens in need of care and treatment, approaches that foster more efficient decision-making and treatment processes are becoming imperative to maintain comprehensive care. Thus, an AI-enabled CDSS represents an important and future-oriented measure that enables actors in the health care domain to improve resource allocation, make timelier and less stressful decisions, and cope with shortages in personnel, facilities, and expertise. However, the potential application of CDSSs and pursued benefits calls for investigations that shed light on how AI-enabled processes can be implemented within prevalent health care structures so that the associated risks and challenges, such as the oversimplification of individual patient data or the automated initiation of suboptimal or erroneous treatments, can be mitigated.

## Appendix

Multimedia Appendix 1 Documentation of full-text exclusion.

## Abbreviations

AI: artificial intelligence
CDSS: clinical decision support system
PRISMA: Preferred Reporting Items for Systematic Reviews and Meta-Analyses
